# Beggar thy neighbour or befriend thy neighbour? Asymmetric spillovers of China’s double world-class policy

**DOI:** 10.1371/journal.pone.0351226

**Published:** 2026-06-12

**Authors:** Jianqin Hang, Xing Ma

**Affiliations:** 1 School of Economics and Management, Jiangsu Maritime Institute, Nanjing, China; 2 Research Center for Language and Language education, Central China Normal University, Wuhan, China; 3 Higher Education Institute, Nanjing University of Information Science & Technology, Nanjing, China; CIFE Mexico, MEXICO

## Abstract

Higher education policies commonly engender certain spillover effects, which frequently remain unaccounted for within the formal policy evaluation framework. Focusing on China’s Double World-Class (DWC) strategy, this paper investigates, from both theoretical and empirical perspectives, whether and how the policy is associated with the research output of non-selected disciplines. Theoretically, this paper puts forward two possible causes of spillovers: reputation enhancement and resource dilution. Based on discipline-level empirical data in Chinese universities from 2014 to 2023 and combined with a panel-regression model, this paper examines whether this policy is associated with changes in the quantity and quality of research outputs among Chinese- and English-language publications across different areas. The results show that the World-Class Discipline (WCD) policy is associated with considerable asymmetric spillover effects on non-selected disciplines. This policy variable is associated with a reduction of 0.62% in the quantity and 1.77% in the quality of research outputs published in Chinese core journals in non-selected disciplines. Meanwhile, it is positively associated with an increase of 5.57% in quantity and 5.06% in quality of research outputs published in English journals. The results of mechanism tests indicate that the enhancement of university reputation and the dilution of educational resources play important roles in the asymmetric spillover effects of WCD policy. The heterogeneity analysis results show that the spillover effects of this policy change across different regions and disciplinary areas. Additionally, this paper integrates the Socialformation Paradigm theory and the Sustainable Social Development theory to further discuss the “delocalization” findings of WCD policy.

## 1. Introduction

In the contemporary university education system, the discipline constitutes one of the fundamental units for both undergraduate education and scientific research. The development level of disciplines not only affects the competitiveness of an individual university but also profoundly influences a nation’s overall innovation ecosystem and international scientific standing. To enhance national competitiveness in research and education, many governments have introduced major reform policies aimed at cultivating world-class universities and elevating scientific “excellence”. For instance, Germany’s “Excellence Initiative” has significantly influenced disciplinary selection and resource allocation patterns through competitive funding [1]. Amidst the global wave of higher education reform, WCD (World-Class Discipline) has gradually evolved into a policy model centered on research output, talent aggregation, and global reputation building. At its core, this model treats high-quality research and international comparative performance as the primary yardstick for evaluating the effectiveness of educational policies [2].

Evaluating the consequences of higher educational reform policies has become an important issue for research on higher-education administration. Many existing higher-education policy studies focus solely on their impacts on selected universities or disciplines [3,4], while ignoring those on non-selected universities or disciplines. If there is a negative spillover effect on other research areas in the same department due to the increase in scientific contributions of WCD, then the actual policy results obtained at this level will be overstated. Therefore, this paper proposes incorporating spillovers into the policy-evaluation framework to provide a theoretical basis for avoiding inaccurate evaluation. As interdisciplinarity is becoming more prevalent, examining the spillovers of discipline-development policies in higher-education research needs to be included as part of policy evaluation.

Internationally, academia has developed a relatively systematic discourse on the structural consequences of “excellent-oriented” research policies. Since Germany’s “Excellence Initiative” was implemented, an increasing number of countries have adopted competitive selection and concentrated resource allocation to cultivate a small number of “centers of excellence” in order to enhance global research competitiveness. However, some studies suggest that while strengthening the performance of top institutions, this approach also creates a stratified structure within higher education systems and between institutions [5]. Germany’s Excellence Initiative leverages competitive funding allocation to reinforce resource disparities within the higher education system. Funded universities significantly outperform non-funded institutions in securing third-party funding and other resources, demonstrating how excellent-oriented policies exacerbate existing inequalities and redistribute educational resources [6]. Similarly, research on performance-based research funding systems (PRFS) reveals that evaluation systems not only alter research output structures but also influence organizational strategic behaviors and disciplinary alignments, exhibiting systemic impacts beyond directly funded units [7]. Although existing literature has revealed the profound effects of elitist policies on systemic stratification and organizational behavior, most studies focus on funded institutions or overall system performance changes. Detailed micro-level evidence remains scarce regarding whether spillover effects exist between different disciplines within the same organization—particularly how reputation transmission and resource restructuring interact.

The DWC (Double World-Class) policy, introduced by the Chinese government in 2015 to build world-class universities and disciplines, has attracted widespread attention among scholars [8–10]. This policy falls under the category of “excellent-oriented” higher education reform, aiming to support selected universities and disciplines in enhancing their competitiveness and influence within the global higher education system, thereby advancing China’s higher education as a whole toward world-class standards. It builds upon China’s earlier key development initiatives like the “Project 211” and “Project 985”, while incorporating dynamic adjustment mechanisms, performance-based funding, and international competitiveness orientation into its policy logic—differing from previous identity-based funding models. The DWC policy has more typical manifestations of a structure compared with earlier key construction projects. Firstly, policies will no longer cover every field at all universities but rather focus on specific disciplines within those institutions at different levels. That is to say, resources are concentrated in some fields and not spread out among various departments of the university. Secondly, the dynamic adjustment mechanism and the periodical assessment system strengthen the performance-oriented competitive orientation of selection to prevent long-term stability from being affected by research outcomes or other factors such as international comparisons. Third, current resource allocation includes both centralized funding and competitive grants to distribute funds more efficiently to strong areas. Although this kind of arrangement can improve the ability to develop in specific fields, it will reshape the situation regarding resource distribution and interdisciplinary relationships at that university, creating an institutional context that correlates with similar changes across various academic domains.

Even though the tendency towards excellent-oriented policies in world’s top universities has taken shape now, most research thus far focuses only on how those broad policies affect one institution or one academic field. There are few studies yet into the different impacts within an organization. Under the institutional conditions of highly quantifiable research evaluation and ranking-oriented incentive mechanisms, resources become unevenly distributed among universities that shape the competitiveness structure and channel distribution paths within organizations [5]. However, under the framework of excellence policies for higher education, systematic micro-level empirical research on these intra-organizational activities’ effects is currently scarce. China’s DWC policy shares significant institutional similarities with these international reforms, making it suitable for examining the indirect impacts of excellent-oriented policies.

Given the aforementioned gaps in the literature, this study focuses on examining the spillover effects of the WCD initiative on non-selected disciplines within universities. Theoretically, the policy may influence non-selected disciplines through two opposing mechanisms. First, disciplines that receive this world-class evaluation boost the overall academic recognition and prestige of related universities’ systems. Therefore, a university’s ranking of world-class universities can serve as an external quality indication, increasing opportunities and recognitions for other disciplines in research cooperation, talent attraction and academic publications at this institution, thus producing a reputation spillover effect. Conversely, distributing resources of the concentration policy in particular fields leads to imbalances among these areas; other unrelated disciplines may thus suffer as well. Interaction among this pair brings about uncertainty concerning the direction of policy spillovers.

Therefore, systemically investigating the spillover effect of the WCD policy on non-selection disciplines from theoretical to empirical perspectives is worthy of our attention. Theoretically, these three kinds of theories are all involved: unbalanced growth [11], group reputation theory [12], and the marginal equilibrium analysis by Marshall [13]. Empirically, this paper uses discipline-level data to test whether academic output quantities and qualities across disciplines exhibit “beggars-thy-can-believe-it” (beggar-thy-neighbour) or mutual benefits phenomena.

This paper intends to explore the following issues. The following issues are intended to be explored. Does the WCD policy have a transmission effect on other fields outside of those chosen? Second, are the spillovers beneficial or costly? Third, how does the policy generate spillover effects across non-selected fields?

The structure of this article is as follows: Section two, policy background and literature review; Section three presents theoretical framework and hypotheses; Section four outlines research design; Section five reports empirical results; and Section six concludes with findings and policies’ implications.

## 2. Policy background and literature review

### 2.1 Policy background

DWC is an important new higher education reform plan initiated by China after 2015, which has been promoting college reforms across the country. To boost the global competitiveness of Chinese higher education through targeted innovations, it supports China’s innovation-driven development strategy [9]. The DWC policy represents both the inheritance of China’s post-1949 educational reform policies and an enormous change in higher education governance systems. The earlier key-development mode focused primarily on identity-based funds; compared with earlier policies, the DWC initiative has introduced multiple new components of institutions. First, the “Overall Plan for Coordinated Advancement of World-Class Universities and Disciplines” and its implementation rules establish disciplines as the fundamental unit for allocating higher education resources—a significant departure from the previous practice of treating only universities as a whole as key development targets. Second, the policy institutionally introduces a dynamic adjustment mechanism with both entry and exit, enabling warnings or revocation of construction qualifications to break the rigid identity framework. Additionally, the Evaluation Measures for the Construction of DWC Universities and Disciplines establishes a multi-dimensional performance evaluation system, using evaluation outcomes as the key criterion for retaining construction qualifications. Concurrently, the policy requires that evaluation of construction outcomes be oriented toward global scientific frontiers and international advanced standards, meaning internationally comparable indicators and global competitiveness have become important references in policy design. This institutional logic shows significant similarities to excellence initiatives implemented by many countries worldwide. Exemplified by Germany’s long-standing Excellence Initiative, such excellent-oriented policies for research and talent cultivation aim to enhance the overall competitiveness and international visibility of the national research system through competitive funding support, thereby elevating the standing of domestic universities in the global higher education market.

As summarized in Table 1, China’s excellence initiatives have evolved through four stages. The first stage (1949–1965) focused on designating key universities such as Renmin University, Peking University, and Tsinghua University, emphasizing institutional rather than disciplinary development. The second stage (1976–1986) restored and expanded the system, increasing the number of key universities to 88. The third stage (1995–2015) introduced the “211 Project” and “985 Project”, concentrating resources on selected universities to build world-class institutions, yet also reinforcing institutional stratification and Matthew effects [14]. The current DWC stage (2015–present) replaced the static identity-based model with a performance-oriented, discipline-focused mechanism, introducing dynamic adjustment of support. The first and second lists of supported institutions were released in 2017 and 2022, respectively.

**Table 1:** The implementation stages and characteristics of major policies in Chinese higher education.

Stage	Landmark Policies/Projects	Main characteristics	Focus on University construction	Focus on disciplinary construction	Dynamic adjustment
Key university Construction (1949–1965)	Resolution on the Work Scope of Key Universities and Experts (1954)	Establishment of the first batch of 6 key universities, focusing on overall university construction	Yes	No	No
Restoration and expansion (1978–1986)	Report on Restoring and Developing National Key Universities (1978)	Number of key universities increased to 88	Yes	No	No
Engineering construction (1995–2015)	211 Project (1995)、985 Project (1998)	Focus on constructing 100 key higher education institutions	Yes	No	No
DWC construction (2015-present)	Overall Plan for Coordinated Promotion of World-Class Universities and First-Class Disciplinary Construction(2015)	Discipline-based, dynamic adjustment, performance-oriented	Yes	Yes	Yes

The core of the DWC initiative focuses on leveraging disciplines rather than the previous approach of investing in universities as a whole. It employs a dynamic adjustment mechanism with entry and exit points, coupled with a performance-based incentive system. This model’s advantage lies in significantly enhancing the precision and utilization of resource allocation, stimulating the endogenous motivation and competitive vitality for continuous development among universities and disciplines. It has shifted China’s higher education from scale expansion to quality enhancement, using leading disciplines as a breakthrough point to elevate international competitiveness in scientific research. According to the aforementioned research findings, under an overall planning and implementation scheme of all relevant policies combined with measures for improving college discipline strengths throughout multiple sectors such as systems reform [4,10].

### 2.2 Literature review

Beginning in the 1980s, a large-scale wave of policy reforms has been occurring globally in higher education to improve efficiency, quality, international competitiveness and social service functions. These reforms target in university organizational structures, resource allocation efficiency, research results and social function impacts, which will continue to be examined after these reforms take place. Based on prior studies, it can be inferred that the effects of reform policies focusing on adjusting resource allocation within universities are not always certain [15,16]. Yaisawarng and Ng [15] suggest that China’s resource-oriented “Project 211” initiative has raised the overall quality of some participating institutions to an extent compared to previous levels. However, it still makes up a relatively large proportion among all university rankings. Rutherford and Rabovsky [16] conduct empirical research examining how policies affect 500 higher education institutions across all of America’s fifty states. However, based on their findings, the former’s positive impact is limited at best and potentially harmful after a certain period, demonstrating that policy reliance on only economic incentives has deficiencies in improving higher education quality. These studies indicate that while resource concentration may improve performance for some entities, it simultaneously alters the overall resource distribution pattern within the higher education system.

In recent years, higher education policy research has concluded that excellent-oriented reforms serve not only as a means to enhance individual institutional performance but also as a mechanism for redistributing institutional resources. Competitive funding and elite institution cultivation policies drive research outcomes by selectively supporting certain universities and disciplines. However, such resource concentration inevitably generates a series of consequences. On one hand, national excellence policies like Germany’s Excellence Initiative rely on competitive funding allocation to boost output at selected institutions. Yet from a quality metrics perspective, their effects are complex and exhibit negative feedback [17]. On the other hand, these policies globally drive universities to reshape their international rankings and research competition landscapes, concentrating resources toward dominant entities [18]. From a perspective of education institution theory, the logic of resource concentration and competitive-oriented policies can enhance the competitiveness of elite units but also create Matthew effect and system imbalance problems within the system. This forms a theoretical basis for exploring the structural effects of excellence policies.

Higher educational administrative policy reforms’ influences still need confirmation through further studies. Agasisti and Dal Bianco [19] concluded that after introducing the BA-MA system within their teaching reforms in Italy for some time, there was an improvement in teaching effectiveness. However, at first, this affected work performance negatively. The implementation of new-managerial reform in European universities has produced some unexpected effects. For instance, the quantitative evaluation orientation in research has fostered a tendency toward formalization in social services, while conflicts between academic autonomy and neo-managerialism have emerged within governance structures [20]. Differences in political-administrative systems effectively explain variations in the implementation outcomes of higher education reform policies across European countries [21]. While university enrollment expansion policies met societal demands, they also exacerbated educational resource shortages and equity issues [22]. Against the backdrop of globalization, internationalization has become a primary trend in higher education reform. Research on China’s top universities indicates that scholars returning from overseas play a crucial role in enhancing internationalization performance [9].

As the latest policy in China’s higher education reform, the DWC Initiative is characterized by discipline-based selection, performance-driven incentives, reform-driven momentum, and dynamic adjustment. Its impact has become a hot research topic in recent years. Empirical studies have confirmed the policy’s positive effects in enhancing research output and overall competitiveness among selected universities. Table 2 shows the representative studies on the impacts of China’s DWC Policy. Ma and Luo [3] employed a DID model to analyze 39 first-class university construction institutions and 57 first-class discipline construction institutions, confirming that the policy produced statistically significant positive impacts on comprehensive strength, discipline development, faculty quality, and social service capabilities. Chen et al. [23] found that DWC universities exhibit higher overall resource allocation efficiency, with changes in technical efficiency and technological progress jointly driving improvements in total factor productivity.

**Table 2:** Representative studies on the impacts of China’s DWC policy.

Impact dimension	Key findings	Representative studies	Core effect	Study samples
Overall research and competitiveness	Enhanced research output and competitiveness of selected institutions	[3,23]	Positive	University
Disciplinary research development	Significant gains in chemistry and biology; stronger for comprehensive universities	[4]	Positive, heterogeneous across disciplines	Discipline
Student quality	Raised admission scores, particularly in science, agriculture, medicine, and niche disciplines	[10]	Positive	University
University governance	Resource concentration, selective neglect, and policy decoupling	[24,25]	Mixed, often negative	University

The DWC Initiative relies on disciplines as its foundation, with varying outcomes across different fields. Using the differences-in-differences (DID) approach, Zhu and Chen [4] find evidence that this policy increases its researchers’ output in some related topics, while having negligible or negative impacts elsewhere. Overall university benefits most significantly under this measure, compared with other forms of institution. Zhu and Zeng [10] find that the DWC policy increases entrance examination scores at some universities’ corresponding departments by enhancing the institution’s credibility. It works especially well to enhance the quality of student recruitment in science, agriculture, medicine, and traditional fields with low appeal. The policy has shown a certain effect on improving disciplinary image and drawing talent.

However, although the DWC policy has raised the level of research in universities and disciplines, there have been problems such as lack of resources and a separation between policies. Based on the case study of a provincial university selected to participate in the DWC project, the policy had some unexpected effects such as selective neglect and too much investment in certain fields, which affected higher education quality [24]. In addition, universities show a convergence in their DWC goals. On the one hand, they standardize organizational structure and practice to obtain government resources [25]. On the other hand, they pursue differentiated positioning amid fierce competition to leverage their strengths, leading to “policy decoupling”. This phenomenon occurs when these policy responses diverge from the actual operations of their academic units [8].

Although existing research has generally focused on the impact of excellent-oriented policies on their beneficiaries, there remains limited attention to the indirect effects these policies generate within organizations. Under education policies, particularly excellent-oriented frameworks, dominant disciplines gain developmental opportunities through resource allocation. The accompanying reputation boost may spill over to other disciplines. However, resource concentration may simultaneously squeeze non-selected disciplines in terms of research funding, talent attraction, and internal support. Therefore, examining spillovers from the perspective of potential positive and negative impacts of “excellence units” on non-selected entities within organizations is a crucial direction for deepening the understanding of policy effects. However, systematic empirical testing of this in existing research remains scarce.

At present, while existing literature has broadly addressed the role of excellent-oriented policies in driving research performance and international competitiveness, both theoretical and empirical perspectives reveal areas warranting further exploration. First, most studies focus on the “direct beneficiaries” of such policies, emphasizing performance improvements in selected universities or dominant disciplines, yet they lack systematic analysis of potential structural redistribution effects within organizations. However, resource competition and cumulative advantage theories suggest that selective resource allocation creates new internal imbalances within organizations [26]. Second, existing studies predominantly examine universities as holistic entities, neglecting the role of disciplines as core units for resource allocation and performance evaluation. Third, while existing studies indicate that excellence policies intensify research competition, direct empirical verification is lacking regarding specific operational pathways—such as whether they drive overall disciplinary development through reputation spillover effects or cause relative weakening of other disciplines via resource reallocation. Therefore, this paper takes a micro disciplinary perspective and combines internal and external organizational spillovers in the analysis system. Based on empirical models of reputation spillback and resources dilution mechanism, it will systematically investigate the effects of WCD policy and deepen existing theoretical explanations through empirical methods.

## 3. Theoretical framework and research hypotheses

The policy to build WCD is an essential part of the DWC plan. It is the process of aggregating strong resources to build WCD by non-equilibrium resource allocation and competition. The policy logic originates from the “theory of uneven economic development” in development economics, assuming that under resource limitations, focusing on the development of promising “development poles” can promote overall advancement via diffusion effects [11]. Based on Hirschman’s [27] “Unequal Growth” theory, it is a result of the so-called spilldown. The selection procedure tends to generate an uneven distribution and attract adverse effects on society [28]. Based on Marshall’s externality theory [13], this paper believes that the policy will have dual effects on non-selected fields under both positive and negative forms of externalities respectively. Two competing hypotheses result from this:

H1a: The WCD policy brings positive spillovers to non-selected disciplines through improved research outcomes.

H1b: The WCD policy correlates with adverse effects on non-selected disciplines through restrictions on their research outcomes.

The WCD policy focuses resources to nurture a small number of outstanding sectors. Its institutional logic is essentially an unbalanced development strategy towards excellence. In this institutional set-up, the policy exerts an impact on the development of non-selected disciplines via two separate but opposing mechanisms: Firstly, it enhances the entire university’s reputation through quality indicators and organizational power to produce a positive spill-over effect. Secondly, it reallocates resources and changes institutions’ assessment frameworks to lead to negative crowding-out consequences for within-school relationships of resource dependence and researcher decision-making processes. The two paths are intertwined to produce an uncertain net policy-spillover effect.

Reputation serves as an indispensable and unseen resource for discipline-building work [10]. Based on the mutual dependence of reputation at both individual and group levels under collective-reputation theory, the stronger the group recognition, the smaller the impact it has on individuals’ recognition. When the group’s reputation declines, individual’s sense of self-discipline weakens because they fear being excluded, which enters a “degraded reputation-weakened motivation” spiral [12]. Disciplinary reputation at universities serves as a base for institutional prestige. If a subject is selected to enter the WCD list, it may improve the overall image of the institution and benefit non-selected disciplines as well. University reputations have an in-depth impact on research development. On the other hand, a good reputation can attract more excellent students and improve the research level of institutions [10 29,30]. In addition, reputation has an inertia effect that makes it tend to last longer after being built up [31]. It helps continuously attract excellent educational resources to promote improvements in quality and maintain this positive feedback mechanism. We therefore hypothesize:

H2: The policy generates positive spillover effects for non-selected disciplines by enhancing institutional reputation, improving student quality, and strengthening research potential.

In addition to reallocating the entire stock of resources, this policy adjustment in university organizations has affected their strategic decisions on how to distribute limited resources among departments or colleges after losing selected status. According to the view of resource dependence theory that an organization can only succeed under certain external environments, it needs access to a basic commodity and raw material [32]. Institutional environment and resource allocation system prioritize international research indicators. Therefore, non-selected disciplines actively adjust their research directions to improve the quantity of resources obtained and organizational status. They direct more resources into publishing in English-language journals to satisfy the institutional assessment requirements. This is not based on pursuing fame, but a rational reaction to changes in the resource environment.

At this time, the policy will reshape intra-institutional resource allocation. In terms of economy, educational resources are essentially scarce; when we allocate them for education, other aspects have to be curtailed. Based on the marginal and equilibrium analysis for allocating resources at higher returns to investment levels [13], university participants in the WCD initiative will focus their scarce development resources on certain fields and reduce the intensity of resource allocations for other areas. Many local universities, under the impetus of policies, have neglected non-selected disciplines [24], resulting in a waste of resources and eventually affecting the structure of different disciplines’ allocation of resources, resulting in severe negative externalities (negative spillovers) for the non-first-class discipline from a dispersal and squeezing effect. Hence, we hypothesize:

H3: The WCD policy changes the research funding orientation and resource access channels for non-selected fields through adjustments to resource distribution and institutional assessment mechanisms, thus correlating with reduced domestic research output among these disciplines.

Therefore, the WCD policy is associated with spillover effects in opposite directions for non-selected disciplines through reputation enhancement mechanisms and resource restructuring mechanisms. The former emphasizes the dissemination of quality signals and prestige at the organizational level, believing it will increase opportunities for research collaboration and thereby improve the quality of research outcomes. The latter focuses on resource allocation biases and the guiding role of institutional evaluations, leading to localized disciplinary behavior and reduced investment in local research. The relative strength of these two mechanisms determines the final sign of the policy’s spillovers.

## 4. Data and methods

### 4.1. Econometric model

To empirically examine the spillover effects of the WCD policy on research outputs of non-selected disciplines, this study specifies the following empirical model:


$$\begin{array}{c} Spillover_{i,t}=\alpha_{\text{0}}+\alpha_{\text{1}}WCD_{t}+\sum \alpha_{i}Control_{i,t}+\gamma_{i}+\lambda_{t}+\varepsilon_{i,t} \end{array}$$ (1)

where, $Spillover_{i,t}$ represents the research output of discipline i at year t that is not included in the WCD list. This encompasses four dimensions: the quantity and quality of publications in Chinese journals, the quantity and quality of publications in international English-language journals. $WCD_{t}$ is a dummy variable for the WCD construction policy. This policy dummy variable takes the value of 1 if the university where Discipline i is located has any discipline included in the WCD list; otherwise, it takes the value of 0. $\alpha_{\text{1}}$ captures the spillover effect of the WCD policy on the scientific research activities of non-selected disciplines. $Control_{i,t}$ represents a set of control variables. $\gamma_{i}$ denotes individual fixed effects, and $\lambda_{t}$ denotes time fixed effects. $\varepsilon_{i,t}$ stands for the random error term.

### 4.2. Variable selection and measurement

#### 4.2.1. Core explanatory variable.

Following Zhu and Chen [4], the key explanatory variable is operationalized as a binary policy indicator, taking the value of 1 if a discipline’s host university has at least one WCD-selected discipline and 0 otherwise. The WCD list was officially announced on 20 September 2017, marking the formal implementation of China’s new strategic focus on WCD. Accordingly, a policy dummy is constructed for each discipline based on the timing of the initiative. Detailed variable definitions are summarized in Table 3.

**Table 3:** Variable definitions.

Variable type	Variable	Definition
Dependent variable	domestic_Qty	The quantity of publications in Chinese journals
	domestic_Qual	the quality of publications in Chinese journals (Entropy weight method)
	international_Qty	the quantity of publications in international English-language journals
	international_Qual	the quality of publications in international English-language journals (Entropy weight method)
Core explanatory variable	WCD	a dummy variable indicating whether any discipline within the same university has been selected for the WCD. Specifically, the variable takes a value of 1 if the institution hosting discipline i has at least one discipline selected for the WCD List, and 0 otherwise
Control variables	PhD	doctoral student enrollment
	dis_age	discipline age
	funds	the number of national-level projects awarded (including National Natural Science Foundation of China and National Social Science Foundation grants)
	database	expenditure on digital resources
	postgraduate	institutional size (graduate enrolment)
	budget	annual budget
	education	municipal education expenditure
	population	resident population
	pgdp	per capita GDP
Mechanism variables	fame	university reputation expressed as the inverse of its ranking
	PhD_prop	percentage of doctoral admissions in the discipline relative to total admissions

#### 4.2.2. Dependent variables.

To evaluate the impact of the policies, this paper builds a four-dimensional framework to grasp both the quantity and quality of Chinese and English journals’ publications: (1) The number of publications in Chinese journals is measured by the number of articles reported in the “Annual Report on Impact Factors of Chinese Academic Journals”; (2) The number of publications in international journals is calculated through the records in Web of Science (WoS); (3) The quality of publications in Chinese journals is evaluated by bibliometric indicators including high-cited papers, popular papers, their respective proportions, Q1/Q2 publications and their proportions, as well as the average impact factor of each paper; (4) The quality of publications in international journals is measured by high-cited and popular papers, the number and proportion of Q1/Q2 publications, domain and journal standardized citation impact, and the proportion of papers ranked in the top 1% by citation count. An overview of all indicators is provided in Table 4. Composite indices for research quality are generated using the entropy weighting method. Indicators are first normalized to a [0,1] scale, probabilities are calculated, entropy values are derived, and weights are assigned accordingly to compute a weighted aggregate measure of research quality.

**Table 4:** Measurement system for research output indicators.

Primary indicators	Secondary indicators
Scientific research output in Chinese journals	Number of papers published in the Chinese Academic Journals Impact Factor Annual Report
Scientific research output in international English journals	Number of papers in Web of Science
Quality of scientific research output in Chinese journals	Number of highly cited papers in core Chinese journals
	Number of hot papers in core Chinese journals
	Percentage of highly cited papers in core Chinese journals
	Percentage of hot papers in core Chinese journals
	Number of Q1 papers in the Chinese Academic Journals Impact Factor Annual Report
	Number of Q2 papers in the Chinese Academic Journals Impact Factor Annual Report
	Percentage of Q1 papers in the Chinese Academic Journals Impact Factor Annual Report
	Percentage of Q2 papers in the Chinese Academic Journals Impact Factor Annual Report
	Average impact factor of papers in core Chinese journals
Quality of scientific research output in international English journals	Number of highly cited papers
	Number of hot papers
	Percentage of highly cited papers
	Percentage of hot papers
	Papers in Q1 journals
	Papers in Q2 journals
	Percentage of papers in Q1 journals
	Percentage of papers in Q2 journals
	Field-normalized citation impact
	Journal-normalized citation impact
	Percentage of papers ranked in the top 1% by citations

#### 4.2.3. Control variables.

Nine control variables are incorporated to ensure robustness, spanning three levels: (1) Discipline level: the number of doctoral students admitted in the discipline this year (PhD), years since the establishment of the discipline (*dis_age*), and the number of nationally approved projects in the discipline this year (*funds*), including the National Natural Science Foundation and the National Social Science Foundation; (2) University level: universities’ expenditure on purchasing digital resources (*database*), university size represented by the number of graduate students admitted this year (*postgraduate*), and university’s budget amount for the year (*budget*); (3) City level: the amount of educational expenditure in the city’s fiscal budget for the year (*education*), permanent population of the city for the year (*population*), and per capita GDP of the city for the year (*pgdp*). Variable definitions are detailed in Table 3.

#### 4.2.4. Mechanism variables.

Two mediators have been added to examine the pathways by which WCD affects non-selected disciplines. University reputation (*fame*) is proxied by the reciprocal of a national university ranking to capture reputational spillovers from selected to non-selected disciplines. Doctoral student proportion (*PhD_prop*) represents the percentage of doctoral admissions in the discipline relative to total admissions. These variables can reveal the mechanism by which policies affect reputation capital and resource allocation.

### 4.3. Data sources

In this study, the observation period is set from 2014 to 2023 in order to capture changes in discipline-level academic output before and after the implementation of the policy. The analysis focuses on non-selected disciplines. Due to the difficulty of obtaining complete institutional-level control variables for all universities, the sample is limited to universities that publicly disclose relatively comprehensive information. Ultimately, the study covers 226 non-selected disciplines within world-class universities, spanning a wide range of fields including science and technology, humanities and social sciences, and economics and management.

Research output data, including the number of English-language publications, are primarily obtained from the Web of Science and the InCites database [Web of Science: https://www.webofscience.com; InCites: https://incites.clarivate.com]. Specifically, in the InCites platform, publication data are retrieved by selecting the corresponding research areas and affiliating institutions. For each university, discipline-level publication indicators are extracted based on the predefined subject categories. The relevant indicators are then downloaded individually for each institution, and the data are compiled into separate spreadsheets before being merged into a unified dataset for subsequent analysis.

Data on Chinese-language publications are obtained from the Chinese Higher Education Research Output Statistics Database [Chinese Higher Education Research Output Statistics Database: https://cdap.cnki.net/cdap/gpk/platformHome], where discipline-level publication counts are retrieved by selecting universities and corresponding subject categories. The retrieved data are downloaded and organized by year and discipline to ensure consistency with the English publication data.

Discipline-level variables, such as doctoral enrolment and discipline age, are collected manually from official university websites and institutional reports. University-level variables, including annual budget and graduate enrolment, are drawn from publicly available education statistical yearbooks and university annual reports. Data on universities’ expenditure on purchasing digital resources come from the website of the Steering Committee for Academic Libraries of China (SCAL). City-level data, including fiscal expenditure on education, resident population, and per capita GDP, are obtained from the China City Statistical Yearbook.

## 5. Empirical results

In the empirical section, this paper reports the baseline regression results, robustness test results, heterogeneity analysis results, and mechanism test results. The baseline regression and robustness checks (Sections 5.1, 5.2, and 5.4) are confirmatory analyses designed to test H1a, H1b, H2, and H3. The heterogeneity analysis (Section 5.3) is exploratory analysis, intended to generate insights for future research rather than to draw definitive causal conclusions.

### 5.1. Baseline regression

Based on the model specified in Equation (1), and controlling for discipline-, university-, and city-level variables, we employ a two-way fixed effects model with both individual and year fixed effects to examine the spillover effects of the WCD construction policy on non-selected disciplines. The baseline regression results are presented in Table 5.

**Table 5:** Baseline regression results.

	domestic_Qty	domestic_Qual	international_Qty	international_Qual
WCD	−0.0062*** (0.0014)	−0.0177*** (0.0029)	0.0557*** (0.0085)	0.0506*** (0.0067)
PhD	0.0003 (0.0004)	0.0017** (0.0008)	0.0050** (0.0024)	−0.0015 (0.0019)
dis_age	0.0029 (0.0026)	0.0053 (0.0054)	0.0149 (0.0159)	0.0049 (0.0126)
funds	−0.0001 (0.0003)	0.0007 (0.0007)	0.0058*** (0.0021)	0.0022 (0.0017)
database	0.0012** (0.0006)	0.0036*** (0.0012)	−0.0007 (0.0035)	−0.0005 (0.0028)
postgraduate	0.0023** (0.0012)	0.0023 (0.0024)	−0.0039 (0.0071)	−0.0085 (0.0056)
budget	0.0008 (0.0008)	−0.0049*** (0.0016)	−0.0055 (0.0046)	−0.0004 (0.0036)
education	−0.0014 (0.0014)	−0.0031 (0.0029)	0.0164* (0.0086)	0.0057 (0.0068)
population	−0.0059* (0.0032)	0.0077 (0.0067)	−0.0033 (0.0195)	0.0020 (0.0154)
pgdp	0.0014 (0.0018)	0.0059 (0.0039)	−0.0094 (0.0113)	0.0034 (0.0088)
Constant Term	0.0077 (0.0352)	−0.1222 (0.0742)	0.0585 (0.2167)	−0.0065 (0.1706)
Sample Size	2260	2260	2260	2260
γ_i_	Yes	Yes	Yes	Yes
λ_t_	Yes	Yes	Yes	Yes
R^2^	0.0592	0.1347	0.2723	0.2330

Note: The values in parentheses are standard error values; ***, **, and * indicate significance at the 1%, 5%, and 10% levels, respectively.

As shown in Table 5, the WCD policy variable shows a statistically significant negative association with both the quantity (−0.0062) and quality (−0.0177) of Chinese journal research output for non-selected disciplines at the 1% significance level. This indicates that after the policy implementation, non-selected disciplines experience pronounced negative spillovers in domestic research output. This result is consistent with Hypothesis H1b, indicating that the WCD policy correlates with the research levels of other disciplines. Concurrently, columns 3 and 4 of Table 5 show that the policy’s coefficients on the quantity and quality of research output in English journals for non-selected disciplines are 0.0557 and 0.0506, respectively, both significant at the 1% level. This demonstrates a significant increase in international research output for non-selected disciplines after policy implementation. This finding is consistent with Hypothesis H1a.

It is worth noting that the research results from both domestic and international sources show opposite trends, indicating that the external effects of WCD policy are not one-sided but rather exhibit structural differences based on different research dimensions. If this policy merely reduces the allocation of resources to non-selected disciplines, then both domestic and international research results will decline. However, the fact that international research results have significantly increased suggests that in a system where the evaluation criteria increasingly favor international indicators, non-selected disciplines will reallocate their limited research resources to domestic and international publication platforms, thereby exhibiting a substitution effect. Therefore, the decline in domestic results is more likely to be due to a structural adjustment in the direction of research investment rather than a passive loss of resources. Overall, the baseline regression results confirm that the WCD initiative has a strong spillover effect on non-selected disciplines. However, for domestic and international research results, these effects are in opposite directions, which is consistent with the two-way possibility proposed in H1a and H1b.

At the discipline level, the number of doctoral candidates is positively related to both high-quality research output in Chinese Journals and publication volume abroad, which are all statistically significant (coefficient 0.0017 at 5%, coefficient 0.0050 at 5%). This indicates that doctoral candidates are an important resource for research findings. Raising the quantity and discipline structure of pursuing a PhD can boost research efficiency at home. Also, the number of national-level research projects (with a positive coefficient of 0.0058) is positively associated with international output at the 1% significance level. That is, teachers’ participation in funds-funded scientific activities leads them to increase international influence and output accordingly. Universities have identified an inverse relationship among factors. That is to say, there is a strong negative effect on research outputs from the increase in graduates majoring outside first-tier disciplines. Graduate admission numbers have steadily risen in recent years. However, there has been a drop in academic performance evaluation criteria for students entering college. Some students with weak academic research abilities are seeking PhDs mainly to reduce employment pressure. The phenomenon that it reduces the atmosphere for learning and research among grad students exists [33]. At the city level, fiscal expenditures of the host cities correlates with the amount of international academic results. Municipal investments in education may be used to enhance selected disciplines with high competitiveness globally.

In the context of the world-class discipline policy, the phenomenon of declining Chinese journal output and increasing English journal output further suggests that, in China, the research activities of non-selected disciplines are undergoing a structural shift towards “delocalization”. On the one hand, university administrators tend to encourage non-selected disciplines to increase the quantity and quality of their publications in international journals, so as to improve the chances of these disciplines being selected in the next round of dynamic selection for world-class disciplines. On the other hand, researchers themselves are inclined to invest their efforts in areas that are more likely to receive international recognition but may have little relevance to local issues, thereby neglecting academic topics rooted in local realities, such as environmental governance, technological development and social problems. This finding aligns with concerns raised in recent higher education research [34], and this phenomenon poses a potential threat to the ability of universities to serve local social development. However, as to how world-class university construction policies should address the tension between globalisation and localisation, some scholars offer possible solutions [35–38]. They propose the concept of sustainable social development, which goes beyond ecological sustainability or the static Millennium Development Goals; it is a development concept centred on the social dimension, pursuing a systemic integration of economic, ecological and socio‑cultural aspects, and aiming to create equitable, inclusive societies that solve local community problems. Integrating this concept into the evaluation system and educational resource allocation mechanism of world-class disciplines can correct the current “delocalization” bias that over-emphasises international indicators while disconnecting from local realities. By effectively guiding non-selected disciplines to revisit locally grounded issues, this approach helps form a disciplinary development model that balances global academic competitiveness with local social service, and enables universities to become key forces in promoting local environmental governance, technological progress, and social development.

### 5.2. Robustness checks

To ensure the reliability of the baseline regression results, several robustness tests are conducted.

#### 5.2.1. Alternative measurement of the dependent variable.

To rule out potential measurement bias, the dependent variable is recalculated using arithmetic averages. Since the quantity of research outputs has only one indicator, this adjustment is applied only to research quality indicators. The results are reported in Table 6. As shown in the second row of Table 6, even after altering the measurement method, the WCD construction policy is still negatively associated with the research quality in Chinese journals and positively associated with the research quality in international English journals for non-selected disciplines. The sign and significance of the coefficients remain unchanged, indicating that the baseline results are robust to potential measurement bias.

**Table 6:** Robustness test results.

		domestic_Qty	domestic_Qual	international_Qty	international_Qual
WCD	Changing the dependent variable		−0.0115*** (0.0042)		0.0507*** (0.0057)
	Changing the policy timing	−0.0062*** (0.0014)	−0.01768*** (0.0029)	0.0557*** (0.0085)	0.0506*** (0.0058)
	Winsorizing extreme values	−0.0049*** (0.0012)	−0.0151*** (0.0027)	0.0548*** (0.0076)	0.0465*** (0.0054)
	Excluding the impact of the pandemic	−0.0049*** (0.0010)	−0.0031 (0.0021)	0.0341*** (0.0062)	0.0105** (0.0049)
	Excluding the impact of the trade war	−0.0036*** (0.0008)	−0.0035** (0.0016)	0.0126*** (0.0048)	0.0055 (0.0038)
	Excluding the 2014 sample	−0.0065*** (0.0014)	−0.0216*** (0.0031)	0.0465*** (0.0089)	0.0456*** (0.0075)
	Excluding the 2023 sample	−0.0047*** (0.0012)	−0.0103*** (0.0027)	0.0567*** (0.0080)	0.0331*** (0.0048)
	Including lagged factors	−0.0035*** (0.0011)	−0.0177*** (0.0028)	0.0031 (0.0044)	0.0268*** (0.0070)
Sample size	Changing the dependent variable		2260		2260
	Changing the policy timing	2260	2260	2260	2260
	Winsorizing extreme values	2260	2260	2260	2260
	Excluding the impact of the pandemic	2260	2260	2260	2260
	Exclude the impact of the trade war	2260	2260	2260	2260
	Excluding the 2014 sample	2034	2034	2034	2034
	Excluding the 2023 sample	2034	2034	2034	2034
	Including lagged factors	2260	2260	2260	2260
R^2^	Changing the dependent variable		0.0663		0.3411
	Changing the policy timing	0.0592	0.1347	0.2723	0.2330
	Winsorizing extreme values	0.0579	0.1342	0.2995	0.2584
	Excluding the impact of the pandemic	0.0592	0.1347	0.2723	0.2330
	Excluding the impact of the trade war	0.0592	0.1347	0.2723	0.2330
	Excluding the 2014 sample	0.0456	0.1498	0.2635	0.2251
	Excluding the 2023 sample	0.0661	0.0574	0.2671	0.2030
	Including lagged factors	0.4735	0.2803	0.8254	0.3404
Control variables		Yes	Yes	Yes	Yes
Individual fixed effects		Yes	Yes	Yes	Yes
Time fixed effects		Yes	Yes	Yes	Yes

Note: The values in parentheses are standard error values; ***, **, and * indicate significance at the 1%, 5%, and 10% levels, respectively.

#### 5.2.2. Adjusting the policy shock timing.

In the baseline analysis, the policy shock is set to September 21, 2017, the date of the first-round announcement of the WCD list. Considering possible implementation lag effects on research outputs, we adjusted the policy shock timing to 2018 for robustness testing. As shown in Table 6: The negative spillover effect on research output in Chinese Journals and the positive spillover effect on international journal outputs are both found to be statistically significant at a certain period later. 5.2.3. Winsorizing extreme values

To reduce the impact of outliers, a 1% fissurization is applied to both the dependent and independent variable datasets. As shown in Table 6, the coefficient, significance level and conclusion of the whole table are still consistent with the base regression. Outliers have no significant effect on this estimation result.

#### 5.2.4. Excluding other major shocks.

During policy implementation, the research output within higher education systems will be affected by economic conditions outside this scope. Isolating non-policy factors to focus research on two key external shocks: the COVID-19 pandemic and the US-China Trade War. First, given the pandemic’s impact on in-person academic exchanges and laboratory work, an exogenous shock variable (set to 1 for 2020 and beyond, 0 otherwise) was incorporated into the regression model. Table 6 reveals that after controlling for the pandemic shock, the direction of the WCD policy’s impact on domestic and international publications for non-selected disciplines remains unchanged. Most coefficients remain statistically significant at the 1% level, with only the significance of domestic journal output quality declining—though its sign remains negative. Second, to examine the potential impact of the U.S.-China trade war after 2018 on international research collaboration, a trade war dummy variable (1 for 2018 and later, 0 otherwise) was introduced for testing. Regression results indicate that even after accounting for external environmental changes like trade friction, most of the policy’s negative domestic spillover effects and positive international spillover effects remain robust. Therefore, the policy effects identified in this study are not driven by macro-environmental changes and exhibit strong exclusivity.

#### 5.2.5. Adjusting the sample interval.

To examine whether the research conclusions are overly reliant on sample data from a specific period, this paper conducts sensitivity analysis by shortening the observation period. Given that the baseline year (2014) exhibits base-period fluctuations and the final year (2023) may contain statistical lags or unresolved policy effects, the sample data for 2014 and 2023 were excluded for re-estimation. Table 6 regression results indicate that after excluding either the starting or ending year, the sign direction and significance level of the model’s primary estimated coefficients remain broadly consistent with the benchmark regression. Regarding domestic output quantity and quality, a clear downward trend persists, while international output continues to grow. Therefore, policies’ effects have been consistent over time.

#### 5.2.6. Excluding the influence of lagged factors.

Given the obvious path dependence in academic production, where previous research accumulation within a particular discipline influences its current output, this study included lagged terms of the dependent variable in the regression equation to eliminate potential inertial interference. Table 6 shows that after adding the lagged terms, WCD still maintains an influence on domestic research output (both quantity and quality) as well as the quality of international research output. Although the coefficient of WCD became less significant in the regression analysis of international research output quantity, its positive sign still exists. This indicates that even after controlling for the inherent inertia of the discipline’s own development, policy intervention remains the main driving factor for the asymmetric changes in the research output structure of non-selected disciplines, thereby confirming the reliability of the previous conclusion.

### 5.3. Heterogeneity analysis

#### 5.3.1. Regional heterogeneity.

This paper further explores potential heterogeneity in the policy’s spillover effects through categorization of non-selected discipline based on their geographical locations and types.

Table 7 reports the test results for regional heterogeneity. WCD policy variable is negatively associated with research quality in Chinese journals but positively associated with the quantity and quality of international publication in eastern China. This is also consistent with non-selected scholars’ moving towards global attention and interdisciplinary clusters [39,40]. Central regions exhibit a severe reduction in both Chinese- and English-journal publication volumes under this incentive; primarily driven by resource concentration (resource crowding), key fields absorb most researchers and funds while others’ capacities decline significantly. In western regions, the WCD policy is not significantly associated with domestic research output in non-selected disciplines. However, it is positively associated with the quantity of research output in international journals. This is primarily because the policy implementation has also provided development opportunities for non-selected disciplines in western regions, enabling them to access certain academic resources and international collaboration opportunities. Consequently, the quantity of research output in international journals has increased significantly. Nevertheless, due to persistent factors such as talent shortages, scarcity of academic resources, insufficient funding allocation, and limited capacity to attract outstanding students, the quality of their research output has not seen a noticeable improvement. Therefore, the spillover effects of the WCD policy on the quantity and quality of research output in domestic and international journals differ for non-selected disciplines.

**Table 7:** The test results for regional heterogeneity.

		domestic_Qty	domestic_Qual	international_Qty	international_Qual
WCD	Eastern China	0.0021 (0.0019)	−0.0093** (0.0042)	0.0581*** (0.0121)	0.0507*** (0.0096)
	Central China	−0.0216*** (0.0054)	−0.0328*** (0.0107)	−0.0936*** (0.0302)	−0.0302 (0.0252)
	Western China	−0.0067 (0.0056)	0.0016 (0.0133)	0.0896*** (0.0331)	0.0111 (0.0257)
Sample Size	Eastern China	1310	1310	1310	1310
	Central China	660	660	660	660
	Western China	290	290	290	290
R^2^	Eastern China	0.0552	0.1710	0.2929	0.2330
	Central China	0.1653	0.1902	0.3872	0.3114
	Western China	0.2019	0.0809	0.3714	0.3018
Control variable		Yes	Yes	Yes	Yes
Individual fixed effects		Yes	Yes	Yes	Yes
Time fixed effects		Yes	Yes	Yes	Yes

Note: The values in parentheses are standard error values; ***, **, and * indicate significance at the 1%, 5%, and 10% levels, respectively.

The phenomenon of increased international publication quantity but stagnant quality in Western China reveals a risk of “low-quality internationalisation” driven solely by quantitative evaluation metrics. The “socioformation” educational paradigm offers a highly promising framework for the construction of world-class disciplines in China [35,37,41]. The value of socioformation lies in anchoring learning objectives in the collaborative transformation of the social environment and the achievement of community sustainability, thereby effectively weakening the tendency to simply equate academic activity with international indicators. Mounkoro [42] further points out that teachers play a key role in the paradigm shift towards socioformation: they need to transform from knowledge transmitters to learning facilitators, coordinators and project designers. The socioformation framework emphasises competence development anchored in community sustainability [43,44], and this developmental model provides a framework for addressing the aforementioned challenge. Reforms of world‑class policies should not merely encourage non-selected disciplines to publish more internationally; they should also incorporate socioformative criteria that reward research addressing local development challenges. Integrating attention to socioformation into the evaluation system of world-class disciplines would enhance the rationality of policy design.

#### 5.3.2. Discipline-type heterogeneity.

Table 8 presents the heterogeneity of spillover effects across disciplines (social sciences, natural sciences, and engineering). The WCD policy is negatively associated with the quantity and quality of Chinese journal publications across all disciplines while positively associated with international journal outputs. This demonstrates that the policy has played a positive role in enhancing the overall development level and international influence of disciplines. The policy provides incentives for non-selected disciplines, encouraging them to build according to the standards required by the policy, concentrate existing research resources, and increase investment to enhance their international influence and competitiveness. Comparing among different disciplinary categories shows that the non-selected engineering disciplines show significantly stronger positive spillover effects on both the quantity and quality of international journal outcomes. These differences stem from variations in the disciplinary knowledge-structure of evaluation system. Research methods, technical specifications, etc., in engineering and natural sciences have strong universality and consistency across countries internationally. Thus, it is more possible for them to achieve recognition in the unified world-class examination system. Due to a good overall reputation of the university as a result of WCD development, engineering disciplines can seize this opportunity and participate in international research networks more easily, thereby enhancing their own benefits. Contrastingly, the research themes of social sciences disciplines are more closely tied to language use and context-specific. The proportion of local knowledge production remains high, while the marginal gains from international publications are modest. Hence, there is less effective policy spillover.

**Table 8:** The test results for discipline category heterogeneity.

		domestic_Qty	domestic_Qual	international_Qty	international_Qual
WCD	Social sciences	−0.0056* (0.0030)	−0.0150** (0.0072)	0.0110*** (0.0027)	0.0401** (0.0157)
	Natural sciences	−0.0067** (0.0030)	−0.0148** (0.0059)	0.0387*** (0.0096)	0.0592*** (0.0135)
	Engineering	−0.0042** (0.0018)	−0.0202*** (0.0037)	0.0853*** (0.0160)	0.0530*** (0.0088)
Sample Size	Social sciences	600	600	600	600
	Natural sciences	710	710	710	710
	Engineering	950	950	950	950
R^2^	Social sciences	0.1816	0.2435	0.4237	0.1470
	Natural sciences	0.0799	0.1385	0.3464	0.2611
	Engineering	0.0714	0.1296	0.4066	0.3512
Control variable		Yes	Yes	Yes	Yes
Individual fixed effects		Yes	Yes	Yes	Yes
Time fixed effects		Yes	Yes	Yes	Yes

Note: The values in parentheses are standard error values; ***, **, and * indicate significance at the 1%, 5%, and 10% levels, respectively.

### 5.4. Mechanism analysis

In order to test the mechanism behind the spillover effect of the WCD policy through empirical research, we examined two paths: (i) the reputation enhancement mechanism and (ii) the educational resource dilution mechanism. University reputation and the proportion of doctoral students in each discipline are selected as mediating variables. The results are presented in Table 9. For the reputation mechanism, the estimated coefficient of school reputation is 0.0040, which is statistically significant, indicating that the WCD policy is significantly positively associated with the overall university reputation. This supports Hypothesis H2. From the perspective of the educational resource dilution mechanism, the estimated coefficient of the mediating variable is −0.0065 and has passed the significance test. This also reflects the scarcity of resources in neoclassical economics theory [45] and the policy concerns proposed by Li and Duan [46], who propose that this policy concern prompts universities to allocate their limited resources (funds, talents, equipment, etc.) primarily to selected disciplines, thereby crowding out investments in non-selected disciplines. This creates a resource siphoning effect that suppresses research activities in non-selected disciplines. This supports Hypothesis H3, indicating a negative spillover effect due to resource reallocation.

**Table 9:** Mechanism testing results.

	Reputation enhancement mechanism	Educational resource dilution mechanism
	fame	phd_prop
WCD	0.0040*** (0.0002)	−0.0065** (0.0030)
Constant term	0.0211*** (0.0045)	0.3309*** (0.0847)
Control variable	Yes	Yes
Individual fixed effects	Yes	Yes
Time fixed effects	Yes	Yes
Sample size	2260	2260
R^2^	0.8117	0.1477

Note: The values in parentheses are standard error values; ***, **, and * indicate significance at the 1%, 5%, and 10% levels, respectively.

Overall, the mechanism analysis reveals a “double-edged sword” effect: while the WCD policy positively affects non-selected disciplines via reputation enhancement, it simultaneously exerts a negative spillover by concentrating educational resources toward selected disciplines.

It is worth noting that the dilution effect of the first-class discipline development policy on educational resources for non-selected disciplines is not immediate. There is often a certain time lag in policy implementation and resource allocation. Therefore, when examining this mechanism, this paper treats the doctoral enrollment share of non-first-class disciplines as one period ahead (i.e., using t + 1 period data as the dependent variable). This approach is dictated by the objective constraint of limited access to discipline-level granular data. The lagged effect better captures the dynamic adjustment process of resource allocation. Moreover, since adjustments to doctoral enrollment quotas are typically influenced by prior policy directives, using a lagged term effectively addresses the issue of mutual influence between the explanatory variable and the mediator variable, thereby mitigating endogeneity concerns to some extent. The regression results in Table 9 indicate that, even after accounting for time lag effects, the coefficient for doctoral enrollment share remains statistically significant.

Although the reputation enhancement mechanism generates positive spillovers, its effect on preventing “delocalization” is limited. In fact, an improved institutional reputation may further incentivise non‑selected disciplines to pursue internationally prestigious publication outlets, thereby potentially intensifying the neglect of local issues. Corresponding to the dilution of limited educational resources, the diffusion of public technologies may also accelerate this asymmetric spillover effect. As a general‑purpose technology, artificial intelligence further exacerbates the delocalization trend. A study on generative AI and educational agency found that while generative AI can enhance learners’ agency through personalised learning support and content adaptation, it may also exacerbate educational inequality and weaken learners’ autonomy in certain contexts [47]. Under the current research evaluation mechanism, AI technology may intensify the academic “delocalization” trend triggered by the world‑class discipline policy. The application of AI accelerates the circulation and promotion of mainstream research agendas, thereby generating paper topics that have weak ties to local problems.

Facing this dilemma, the integration of the socioformation model with artificial intelligence becomes particularly critical. In a study on innovation practices in the IT industry in Mexico and the United States, Pastrana et al. [48] explicitly point out that combining the socioformation framework with AI‑driven tools not only helps identify talent skill gaps and design personalised development pathways, but also mitigates the weakening effect of technology on learner agency while enhancing innovation capacity. Therefore, future DWC policy reforms should not merely adjust resource allocation; they should also explore how to introduce the experience of integrating socioformation and AI into the curriculum and research evaluation reforms of China’s world‑class discipline universities, so as to balance international competitiveness with localised development and achieve sustainable social development.

## 6. Conclusion

The spillovers of higher education policies are often overlooked, leading to inaccurate evaluations of policy outcomes. This paper examines the asymmetric spillover effects of the Chinese government’s DWC program on disciplines that have not been included in the WCD list. Based on a systematic literature review, this paper constructs a theoretical framework and identifies two mechanisms: reputation enhancement and resource dispersion. Using panel data of disciplines in China from 2014 to 2023, this paper establishes a regression model to assess the impact of this policy on the quantity and quality of research outputs in Chinese journals and English journals. This paper also examines the heterogeneity across different regions and disciplines and tests the potential mechanisms. The results show that the DWC policy is negatively associated with the quantity and quality of publications of non-selected disciplines in Chinese journals, while positively associated with the quantity and quality of publications in international journals, presenting an asymmetric spillover effect pattern. The heterogeneity analysis indicates that these effects vary significantly across different regions and discipline types, and the effects are more significant in the central and western regions. The mechanism test shows that the policy has a positive pulling effect by enhancing the reputation of institutions, but it also has a negative crowding-out effect due to the imbalance in resource allocation.

Importantly, the decline in Chinese journal outputs and the increase in English journal outputs point to a structural shift towards “delocalization” of research activities in non-selected disciplines – a trend that may weaken universities’ capacity to serve local social needs (Shu and Tian, 2024). As elaborated in Section 5.1, this delocalization tendency is driven not only by resource dilution but also by evaluation systems that over-emphasise internationally comparable metrics. To address the tension between global competitiveness and local responsiveness, we have introduced the socioformation paradigm (Tobón et al., 2015) and the concept of sustainable social development (Luna-Nemecio and Tobón, 2025) into the interpretation of our empirical findings. Integrating these perspectives into the evaluation system and resource allocation of world‑class disciplines could help correct the delocalization bias and foster a more balanced development model.

Based on these findings, we propose three directions for future research and policy. First, future studies should systematically examine how world-class university policies can be designed to promote sustainable social development, ensuring that universities contribute to local environmental governance, technological progress, and social equity. This requires moving beyond simplistic performance metrics towards a more holistic assessment framework that captures universities’ societal impact. Second, a paradigm shift towards socioformation in talent cultivation and research evaluation should be explored, moving beyond mere adjustments of publication metrics. Concrete steps may include redesigning doctoral training curricula and incorporating community-based problem-solving projects as recognised outputs. Third, the integration of artificial intelligence with the socioformation framework warrants attention: while AI may accelerate delocalization under current evaluation regimes, it can also be harnessed-as shown by Pastrana et al. (2025)-to support personalised learning and mitigate the weakening of learner agency, provided it is guided by socioformative principles. Future reforms should consider embedding these insights into the curriculum and evaluation systems of China’s DWC universities, and pilot programmes that combine AI-driven tools with socioformative assessment could be tested in selected disciplines. By taking these directions, policy-makers can better balance international competitiveness with localised development and ultimately achieve sustainable social development.

## Supporting information

S1 Data Data and introduction. (ZIP)

## Abbreviations

WCD: World-Class Discipline
DWC: Double World-Class
