# Supplementary material for: Beggar thy neighbour or befriend thy neighbour? Asymmetric spillovers of China’s double world-class policy
Source: PLoS One. 2026 Jun 12;21(6):e0351226. doi: 10.1371/journal.pone.0351226 (PMC13262825; doi:10.1371/journal.pone.0351226)
Supplement: S1 Data — (ZIP) [file pone.0351226.s001.zip › S1 Data/data introduction.docx]

***Data sources***

In this study, the observation period is set from 2014 to 2023 in order to capture changes in discipline-level academic output before and after the implementation of the policy. The analysis focuses on non-first-tier disciplines. Due to the difficulty of obtaining complete institutional-level control variables for all universities, the sample is limited to universities that publicly disclose relatively comprehensive information. Ultimately, the study covers 226 non-world-class disciplines within world-class universities, spanning a wide range of fields including science and technology, humanities and social sciences, and economics and management.

Research output data, including the number of English-language publications, are primarily obtained from the Web of Science [Web of Science: https://www.webofscience.com] and the InCites database [https://incites.clarivate.com]. Specifically, in the InCites platform, publication data are retrieved by selecting the corresponding research areas and affiliating institutions. For each university, discipline-level publication indicators are extracted based on the predefined subject categories. The relevant indicators are then downloaded individually for each institution, and the data are compiled into separate spreadsheets before being merged into a unified dataset for subsequent analysis.

Data on Chinese-language publications are obtained from the Chinese Higher Education Research Output Statistics Database [https://cdap.cnki.net/cdap/gpk/platformHome], where discipline-level publication counts are retrieved by selecting universities and corresponding subject categories. The retrieved data are downloaded and organized by year and discipline to ensure consistency with the English publication data.

Discipline-level variables, such as doctoral enrolment and discipline age, are collected manually from official university websites and institutional reports. University-level variables, including annual budget and graduate enrolment, are drawn from publicly available education statistical yearbooks and university annual reports. Universities’ expenditure on purchasing digital resources come from the website of the Steering Committee for Academic Libraries of China (SCAL). City-level data, including fiscal expenditure on education, resident population, and per capita GDP, are obtained from the China City Statistical Yearbook.

**S Table 1. Variable definitions.**

| **Variable Type** | **Variable** | **Definition** |
| --- | --- | --- |
| Dependent variable | $domestic\_Qty$ | The quantity of publications in Chinese journals |
|  | $domestic\_Qual$ | the quality of publications in Chinese journals (Entropy weight method) |
|  | $international\_Qty$ | the quantity of publications in international English-language journals |
|  | $international\_Qual$ | the quality of publications in international English-language journals (Entropy weight method) |
| Core explanatory variable | $WCD$ | a dummy variable indicating whether any discipline within the same university has been selected for the WCD. Specifically, the variable takes a value of 1 if the institution hosting discipline $i$ has at least one discipline selected for the WCD List, and 0 otherwise |
| Control variables | $PhD$ | doctoral student enrollment |
|  | $dis\_age$ | discipline age |
|  | $funds$ | the number of national-level projects awarded (including National Natural Science Foundation of China and National Social Science Foundation grants) |
|  | $database$ | expenditure on digital resources |
|  | $postgraduate$ | institutional size (graduate enrolment) |
|  | $budget$ | annual budget |
|  | $education$ | municipal education expenditure |
|  | $population$ | resident population |
|  | $pgdp$ | per capita GDP |
| Mechanism variables | $fame$ | university reputation expressed as the inverse of its ranking |
|  | $PhD\_prop$ | percentage of doctoral admissions in the discipline relative to total admissions |
